# Analysis of Gene Expression Profiles in the Liver of Rats With Intrauterine Growth Retardation

**DOI:** 10.3389/fped.2022.801544

**Published:** 2022-03-07

**Authors:** Zheng Shen, Weifen Zhu, Lizhong Du

**Affiliations:** ^1^Department of Clinical Laboratory, Zhejiang University School of Medicine Children's Hospital, Hangzhou, China; ^2^National Clinical Research Center for Child Health, Hangzhou, China; ^3^Department of Endocrinology, Zhejiang University School of Medicine Sir Run Run Shaw Hospital, Hangzhou, China; ^4^Department of Neonatology, Zhejiang University School of Medicine Children's Hospital, Hangzhou, China

**Keywords:** IUGR, liver, microarray analysis, metabolic syndrome, differentially expressed genes

## Abstract

**Background:**

Intrauterine growth restriction (IUGR) is highly associated with fetal as well as neonatal morbidity, mortality, and an increased risk metabolic disease development later in life. The mechanism involved in the increased risk has not been established. We compared differentially expressed genes between the liver of appropriate for gestational age (AGA) and IUGR rat models and identified their effects on molecular pathways involved in the metabolic syndrome.

**Methods:**

We extracted RNA from the liver of IUGR and AGA rats and profiled gene expression by microarray analysis. GO function and KEGG pathway enrichment analyses were conducted using the Search Tool for the Retrieval of Interacting Genes database. Then, the Cytoscape software was used to visualize regulatory interaction networks of IUGR-related genes. The results were further verified *via* quantitative reverse transcriptase PCR analysis.

**Results:**

In this study, 815 genes were found to be markedly differentially expressed (fold-change >1.5, *p* < 0.05) between IUGR and AGA, with 347 genes elevated and 468 suppressed in IUGR, relative to AGA. Enrichment and protein–protein interaction network analyses of target genes revealed that core genes including *Ppargc1a, Prkaa2, Slc2a1, Rxrg*, and *Gcgr*, and pathways, including the PPAR signaling pathway and FoxO signaling pathway, had a potential association with metabolic syndrome development in IUGR. We also confirmed that at the mRNA level, five genes involved in glycometabolism were differentially expressed between IUGR and AGA.

**Conclusion:**

Our findings elucidate on differential gene expression profiles in IUGR and AGA. Moreover, they elucidate on the pathogenesis of IUGR-associated metabolic syndromes. The suggested candidates are potential biomarkers and eventually intended to treat them appropriately.

## Introduction

Intrauterine growth restriction (IUGR) is highly associated with fetal as well as neonatal morbidity and mortality (1). IUGR is defined as a fetus who does not achieve the expected *in utero* growth potential with a fetal weight <10th percentile (2). IUGR can be caused by various genetic and environment reasons. Studies have reported that IUGR can alter physiological processes and not only is related with elevated risk of short-term complications but also with high risk for developing metabolic syndromes and adverse sequelae in later life, including type 2 diabetes (T2D), insulin resistance, obesity, hypertension, fatty liver disease, short stature, and impaired neurodevelopmental outcomes (3–6). Therefore, IUGR is correlated with an increased risk for adult-onset diseases and is an example of “fetal origins of adult disease hypothesis” by Dr. Barker. The adaptation of the fetus to a deprived intrauterine milieu results in persistent alterations in cellular biology as well as systemic physiology (7). Using several IUGR animal models, the relationship between a high risk of metabolic diseases and fetal growth restriction has been verified (8–10). However, the specific mechanism between IUGR and metabolic syndrome in adult life remains unclear. Clarifying these mechanisms and seeking possible strategies to prevent long-term metabolic effects in IUGR offspring is important for relieving related social–economic burden.

The microarray technology has been widely used to investigate new molecular diagnostic markers, therapeutic targets, and gene functions and evaluate the biochemical pathways to elucidate on disease pathogenesis. This technique is significant in the identification of genes that are vital in the pathogenesis of multifactorial diseases and provides an approach to examine gene expression profiles. Combined with this method, pathway analysis can map gene expression data into appropriate pathway maps based on their molecular associations and functional annotations. By now, a limited number of studies have focused on gene expression patterns in IUGR (11–13). Therefore, we used Affymetrix Gene Chip to evaluate differential gene expression profiling by comparing IUGR newborn rats liver with control. To identify key metabolic syndrome-associated genes, we analyzed their biological roles and associations and lastly illuminated the potential pathogenesis of metabolic disease caused by IUGR.

## Materials and Methods

### Animal Model

Animal assays were conducted at the Laboratory Animal Center of Zhejiang University (Hangzhou, China) in accordance with guidelines in the Guide for the Care and Use of Laboratory Animals of the National Animal Research Center. The Committee on Ethics of Animal Experiments of the Zhejiang University permitted this study (No. ZJU20160215). Prior to surgery, animals were anestheticized using sodium pentobarbital. A total of 25 Sprague–Dawley (SD) rats (17 females and 8 males; weight 250–450 g) were procured from the Laboratory Animal Center of Zhejiang University and housed under normal conditions (room temperature: 18–22°C; circadian rhythm: 12 h light and 12 h dark; humidity: 40–60%). After a week of adaptive feeding, the rats were permitted to mate in a ratio of one male to two females. The beginning of pregnancy was established based on the existence of spermatozoa in the vaginal smears. Twelve rats eventually became pregnant. Rats were fed *ad libitum* with standard SPF Rodent Diet (Shoobree, Nanjing, China), made up of 22% protein, 55% carbohydrates, 4.4% fat as well as 4.1% fiber and could drink *ad libitum* before pregnancy. Pregnant rats were kept in separate cages and randomized into two groups: six pregnant rats as the control group (with initial weights of 273, 270, 268, 293, 252, and 267 g) could still eat and drink *ad libitum*; another six pregnant rats as the IUGR group (with initial weights of 261, 251, 266, 280, 279, and 260 g) could drink *ad libitum* but were restricted to eat and provided with 50% of their typical daily intake from the first day of pregnancy to parturition. After delivery, the weighing of control group neonatal rats was done to calculate the mean values and standard deviations (SDs). AGA was defined if the birth weight of the neonatal rat was between the 10 and 90th percentiles for normal gestational ages. Compared with the AGA group, IUGR rats were identified as those animals with birth weights <2 SDs. The data of the pregnant rats and the corresponding neonatal rats in the control as well as IUGR group are shown in Supplementary Table 1. The pregnant rats delivered spontaneously, and litter sizes were randomly culled to eight per mother at birth, to guarantee the uniformity of litter sizes between control and IUGR litters. Offsprings that did not meet the control and IUGR criteria were culled. Both groups were given plenty of food.

### Tissue Preservation and Total RNA Extraction

We randomly selected five male pups from five pairs of parents in each group. Five offspring in each group came from five different sets of parents. At 1 day of age, these rats were killed with an overdose of sodium pentobarbital. After washing with normal saline, liver tissue samples were harvested and snap—frozen in liquid nitrogen, after which they were stored at −80°C for subsequent uses. Sampling was conducted in the morning, and the interval time was about 10 min.

The total RNA of five IUGR and five AGA rats were extracted from liver tissues using the TRIzol reagent (Invitrogen; Thermo Fisher Scientific, Inc., USA), as instructed by the manufacturer (14). The quality and integrity of RNA were assessed by electrophoresis through 1% agarose gels stained with ethidium bromide. The electrophoretogram of each sample revealed distinct bands corresponding to 18 and 28S ribosomal RNAs. The total RNA from every sample was intact, non-degraded, and adequate for the successive microarray experiments (Supplementary Figure 1). RNA concentrations were spectrophotometrically evaluated on A260 and A280 using NanoDrop 2000 (Thermo Fisher Scientific, Inc., USA). We obtained the A260/A280 ratios of 2.0–2.1 for each sample, which verified the purity of the RNA. Isolated RNA was stored at −80°C for subsequent processing.

### Microarray Analysis

In this assay, the five collected liver tissues from newborn rats with IUGR and five AGA liver tissue samples from rats with normal weights were used as test samples. The total RNA from liver tissues was analyzed using the commercial Affymetrix GeneChip Rat Gene 1.0 ST Array spotted with 27,342 probe sets (Affymetrix, Santa Clara, CA, USA). Affymetrix GeneChip processing and microarray hybridization were performed as previously described (15). In brief, total RNA were converted into double-stranded cDNA using the T7 RNA polymerase (Promega, Madison, WI, USA). After double-strand cDNA generation from first-strand cDNA, the synthesis of biotinylated cRNA was performed by *in vitro* transcription using a BioArray High Yield RNA Transcript Labeling Kit (Enzo Diagnostics, Farmingdale, NY, USA). After the purification of labeled cRNA on RNeasy columns (Qiagen, Hilden, Germany), they were fragmented and thereafter hybridized to the Affymetrix Rat Gene 1.0 Chip (16). This was followed by the hybridization, washing, and staining of the chips. Then, the Affymetrix GeneChip Operating Software (GCOS) was used to generate signal intensities that corresponded to gene expressions. Affymetrix's Expression Console was used for data analysis. Expression values for all samples were obtained by Pathway-level information extractor (PLIER) normalization. Then, the expression values were transformed by log-based two. There might be negative values in the background-adjusted chip data, as well as some single abnormally large (or small) peak (valley) signals (random noise). We excluded negative values and noise signals. The common empirical data- discarding methods included: A: Standard value or singular value discarding method; B: Coefficient of variation method; foreground value <200; Outlook—average/outlook—median <80%, etc. By this way, the elimination of these probe sets reduced the number of false-positive tests. Even though some true-positive values may have been lost *via* this approach, these outcomes were outweighed by eliminations of false positives. IUGR was compared with AGA, and an FDR-corrected *p* < 0.05 was filtered to produce a series of differentially expressed genes (DEGs). Principal component analysis was conducted to evaluate similarities between AGA and IUGR samples (17). Average values were compared to standard values of same samples *via* fold-change filtering. If the value of IUGR group was 1.5 times higher or lower than the control groups' standard value, it was defined as markedly different from the control.

### GO and the KEGG Analyses of the DEGs

The differentially expressed genes (DEGs) list was subjected to gene ontology (GO) terms (http://www.geneontology.org/) and Kyoto Encyclopedia of Genes and Genomes (KEGG) (http://www.genome.jp/kegg/) analysis using the Database for Annotation, Visualization and Integrated Discovery (DAVID) tools (version 6.7; https://david-d.ncifcrf.gov/) to identify GO categories and pathway categories that were overrepresented (18, 19). GO analysis predicted the functions of DEGs in molecular functions (MFs), biological processes (BPs), as well as cellular components (CCs). KEGG was a repository for gene function systematic analysis, linking genomic data with high-level systemic functions from annotation, visualization, and integrated discovery. Finally, in KEGG pathway analyses, overrepresented pathways with *p* < 0.05 were defined as statistically significant.

### Construction of the Protein–Protein Interaction Network

The Search Tool for the Retrieval of Interacting Genes database (version 10.5; http://string-db.org/), which can provide experimental and predicted interaction information (20), was used for the analysis of PPI for DEGs by calculating the combined score. Then, the PPI network of elevated and suppressed DEGs was generated *via* Cytoscape (version 3.2.0; http://cytoscape.org/) (21).

### Verification of Microarray Results by RT-QPCR

To confirm the microarray gene expression data, we performed RT-qPCR to verify the mRNA expressions of six genes. To eliminate possible bias from the sample collection, 16 rat liver tissue samples (eight IUGR and eight AGA) were obtained for RT-qPCR assays. The genes identified for the verification of microarray findings include genes with a potential role in glycometabolism. These genes included peroxisome proliferator-activated receptor gamma, coactivator 1 alpha (*Ppargc1a*), AMP-activated, member 1 (*Slc2a1*), solute carrier family 2 (facilitated glucose transporter), protein kinase, alpha 2 catalytic subunit (*Prkaa2*) (22), retinoid X receptor gamma (*Rxrg*) (23), glucagon receptor (*Gcgr*) (24), and acyl-CoA synthetase long-chain family member 4 (*Acsl4*). Gene-specific primers were designed based on cDNA sequences using the Primer3 software (http://frodo.wi.mit.edu/primer3/), and their sequences are shown in Supplementary Table 2. The extraction of total RNA was performed as mentioned earlier, after which the synthesis of double-stranded cDNA was done using a GoScript Reverse Transcription System (Promega Co, Madison, Wisconsin, USA), as instructed by the manufacturer. RT-qPCR was conducted using the Go Taq qPCR SYBR Green Master Mix (Promega Co, USA) on the ABI StepOne Plus Sequence Detection System (Applied Biosystems; Thermo Fisher Scientific, Inc., Waltham, MA, USA). We performed RT-qPCR experiments with three replicate wells for each sample to enhance the reliability of the findings. Finally, in analysis and graphing, the three replicate wells of each sample were taken as the mean value for statistical calculations. All mRNA expressions were normalized to beta-actin (*Actb*) mRNA levels. The relative expressions for each gene were evaluated *via* the 2^−ΔΔCT^ method.

### Statistical Analysis

Microarray analysis: The normalization of the microarray data was performed using the R/Bioconductor Limma package (25). Microarray gene profiles obtained from five IUGR and five AGA liver samples were analyzed using group comparisons. An empirical Bayes model was used for between-group comparisons of DEGs. Significance was set at *p* < 0.05 and genes with 1.5-fold or greater difference between two groups were obtained for subsequent analyses. We used GO, KEGG, and PPI analyses to assess various features of upregulated and/or downregulated genes from the liver tissue of IUGR rats: the biological processes analysis, biological functional exploration of gene-encoded proteins, and pathway network analysis.

RT-qPCR data analysis: Differences in expression among groups were assessed with Student's *t*-test using SPSS 18.0 software (SPSS, Inc, Chicago, IL, USA). The cut-off for significance was *P* < 0.05.

## Results

### Body Weight Parameters

**Figure 1:**
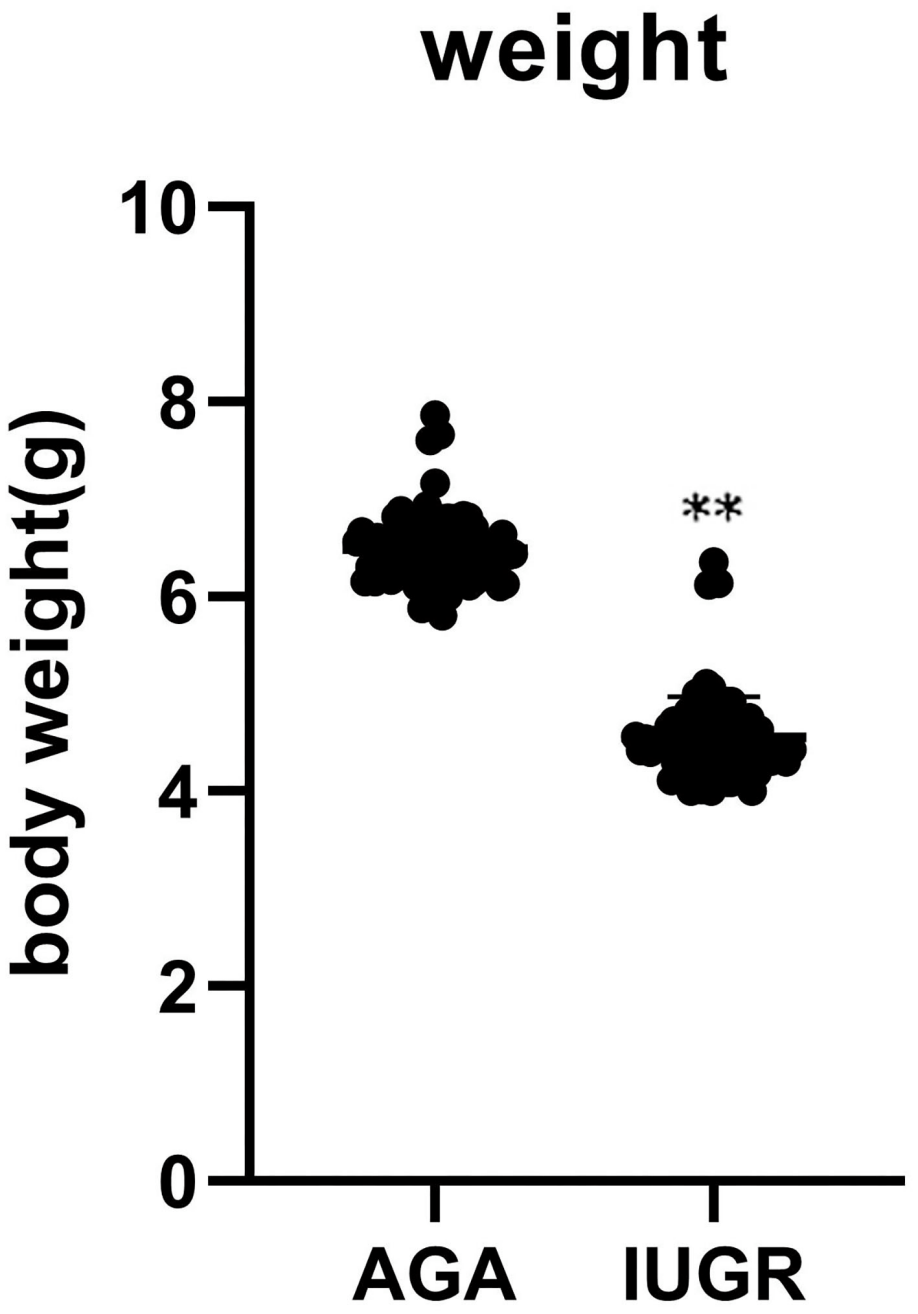
The body weight of 85 AGA (6.49 ± 0.35) and 80 IUGR (4.55 ± 0.42) rats at 1 day (**P < 0.01).

Birth weights at day 1 showed a decrease in average weight by about 30% in the IUGR group, relative to the AGA group (*P* < 0.001) (Figure 1).

### Differential Gene Expression Patterns Between IUGR and AGA

**Figure 2:**
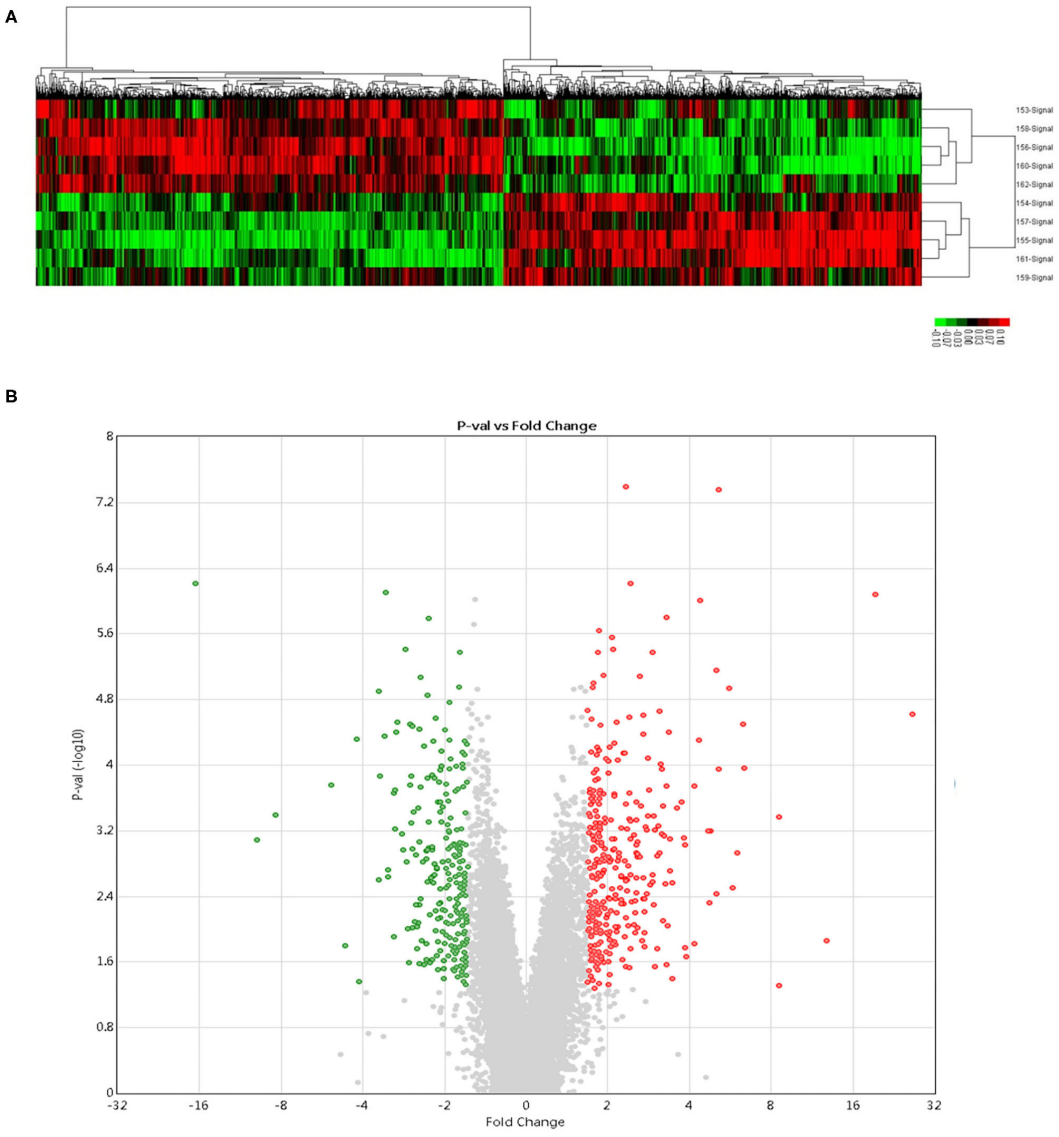
Microarray gene analysis. **(A)** Hierarchical cluster analyses of DEGs. Each probe set is denoted by a single row of colored bars. Red: upregulated; green: downregulated; black: no change. Every line denotes a liver sample from control (*n* = 5) and IUGR (*n* = 5) newborn rats. **(B)** The volcano plot analysis of DEGs. Green color denotes upregulation; Red color represents downregulation. Genes with a significant change of more/ <1.5-fold were selected.

Compared to AGA liver samples, in IUGR liver samples, there were 815 significantly DEGs (*P* < 0.05); among which, 347 genes were elevated while 468 genes were suppressed from the microarray gene analysis. Unsupervised hierarchical clustering of “expressed” genes defined the AGA and IUGR samples (Figure 2A). The volcano plot as well as partial list DEGs were, respectively, presented in Figure 2B and Table 1. Since the analyses were conducted without *a priori* identification of gene groups, it described the real differences in global gene expression patterns, rather than a selection result.

**Table 1:** The top 10 regulated differentially expressed genes between IUGR and AGA.

**Gene symbol**	**Genbank ID**	**Gene name**	**Fold change**	***P*-value**
**Upregulated**				
*Fam134b*	NM_001034912	Family with sequence similarity 134, member B	12.66	5.00E-07
*Upp2*	NM_001106481	Uridine phosphorylase 2	6.10	9.30E-04
*Aspa*	NM_024399	Aspartoacylase	5.22	2.06E-04
*Gadd45b*	NM_001008321	Growth arrest and DNA-damage-inducible, beta	3.49	1.56E-05
*Prkaa2*	NM_023991	Protein kinase AMP-activated catalytic subunit alpha 2	3.47	4.79E-05
*Hbe1*	NM_001008890	Hemoglobin subunit epsilon 1	3.35	2.81E-03
*Ctxn3*	NM_001134696	Cortexin 3	3.27	4.66E-03
*Nr1d1*	NM_001113422	Nuclear receptor subfamily 1, group D, member 1	3.13	1.00E-06
*Gabarapl1*	NM_001044294	GABA type A receptor associated	3.04	4.11E-05
*Cyp2c13*	NM_138514	Cytochrome P450, family 2, subfamily c, polypeptide 13	3.00	1.73E-02
**Downregulated**				
*Inhbe*	NM_031815	Inhibin beta E subunit	0.075	2.99E-05
*Slc34a2*	NM_053380	Solute carrier family 34 member 2	0.077	1.10E-06
*Acot1*	NM_031315	Acyl-CoA thioesterase 1	0.16	1.51E-02
*Cldn2*	NM_001106846	Claudin 2	0.17	1.31E-04
*Cyp4a1*	NM_175837	Cytochrome P450, family 4, subfamily a, polypeptide 1	0.21	1.35E-04
*G0s2*	NM_001009632	G0/G1switch 2	0.21	3.85E-05
*Paqr7*	NM_001034081	Progestin and adipoQ receptor family member VII	0.22	4.95E-04
*Ifi47*	NM_172019	Interferon gamma inducible protein 47	0.22	1.30E-06
*Fam82a1*	NM_001037200	Regulator of microtubule dynamics 2	0.22	1.00E-07
*Paqr9*	NM_001271152	Progestin and adipoQ receptor family member IX	0.23	1.43E-05

### GO Terms of DEGs

**Figure 3:**
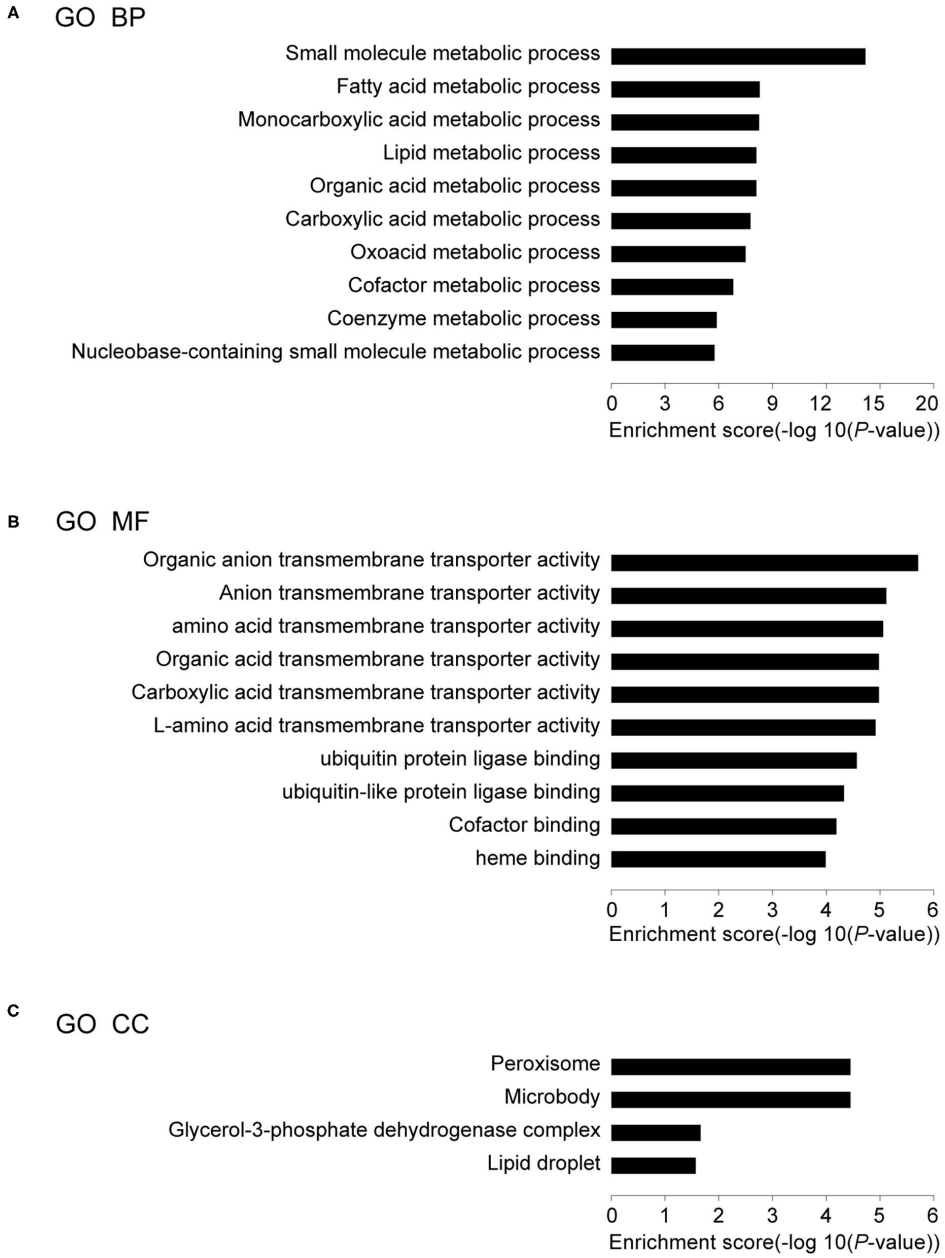
Results of GO analysis. **(A)** Top 10 GO biological processes, **(B)** Top 10 GO MF, and **(C)** Top 4 GO CC in IUGR compared with AGA.

The DEGs in IUGR and AGA were correlated with 30 GO terms in BPs, 4 in CCs, and 46 in MFs. Among the BPs, DEGs were significantly enriched in small molecule metabolic process (59 genes), fatty acid metabolic processes (21 genes), monocarboxylic acid metabolic processes (26 genes), lipid metabolic processes (39 genes), organic acid metabolic processes (34 genes), etc. (Figure 3A; Table 2A), implying that differential expressions of metabolism-associated genes might be involved with development of IUGR-caused metabolic syndrome. With regards to MFs, DEGs were significantly enriched in organic anion transmembrane transporter activity (14 genes), anion transmembrane transporter activity (16 genes), amino acid transmembrane transporter activity (8 genes), organic acid transmembrane transporter activity (11 genes), carboxylic acid transmembrane transporter activity (11 genes), etc. (Figure 3B; Table 2B). The results indicated that transmembrane transporter might be essential for the development of IUGR-caused metabolic syndrome. Moreover, GO CC analysis revealed that the DEGs were markedly enriched in the peroxisome (10 genes), microbody (10 genes), glycerol-3-phosphate dehydrogenase complex (2 genes), and lipid droplet (5 genes) (Figure 3C; Table 2C). The results indicated that cytoplasmic region is involved in the occurrence of IUGR-caused metabolic syndrome.

**Table 2:** The GO terms analysis for DEGs between IUGR and AGA.

**Term**	**Description**	***P*-value**	***N***	**Genes**
**(A) TOP 10 GO TERMS FOR THE BIOLOGICAL PROCESS**				
GO:0044281	Small molecule metabolic process	6.62E-15	59	*Ehhadh, Hao1, Elovl5, Galm, Rgn, Gucy2c, Gldc, Oat, acl1, Abhd1*……
GO:0006631	Fatty acid metabolic process	5.11E-09	21	*Ehhadh, Hao1, Elovl5, Hacl1, Abhd1, Acsm5, Acot5, Acot4, Acot3, Fabp1*……
GO:0032787	Monocarboxylic acid metabolic process	5.53E-09	26	*Ehhadh, Hao1, Elovl5, Hacl1, Abhd1, Acsm5, Acot5, Acot4, Acot3, Fabp1*……
GO:0006629	Lipid metabolic process	5.60E-09	39	*Ehhadh, Hao1, Elovl5, Nrih4, Pnpla2, Hacl1, Agmo, Abhd1, Acsm5, Acot5* ……
GO:0006082	Organic acid metabolic process	7.90E-09	34	*Ehhadh, Hao1, Elovl5, Rgn, Gldc, Oat, Hacl1, Abhd1, Acsm5, Acot5* ……
GO:0019752	Carboxylic acid metabolic process	7.90E-09	33	*Ehhadh, Hao1, Elovl5, Rgn, Gldc, Oat, Hacl1, Abhd1, Acsm5, Acot5* ……
GO:0043436	Oxoacid metabolic process	1.70E-08	33	*Ehhadh, Hao1, Elovl5, Rgn, Gldc, Oat, Hacl1, Abhd1, Acsm5, Acot5* ……
GO:0051186	Cofactor metabolic process	1.51E-07	23	*Rgn, Hbe1, Hbg1, Acsm5, Acot5, Acot4, Acot3, Gpd1, Elovl5, Aldh1l2*……
GO:0015936	Coenzyme metabolic process	1.30E-06	18	*Rgn, Acsm5, Acot5, Acot4, Acot3, Gpd1, Elovl5, Aldh1l2 Acot2, Pank1*……
GO:0055086	Nucleobase-containing small molecule metabolic process	1.73E-06	25	*Gucy2c, Acsm5, Acot5, Acot4, Acot3, Dpys, Nt5c3a, Gpd1, Ppargc1a, Upp2*……
**(B) TOP 10 GO TERMS FOR THE MF**				
GO:0008514	Organic anion transmembrane transporter activity	1.96E-06	14	*Slc25a32, Slc7a2, Slc13a4, Slc25a15, Slc17a2, Slc17a4, Ctns, Slc25a42, Slc25a23, Slc2a1*……
GO:0008509	Anion transmembrane transporter activity	7.52E-06	16	*Slc25a32, Slc7a2, Slc13a4, Slc25a15, Slc17a2, Slc17a4, Ttyh2, Ctns, Slco1b2, Slc25a23*……
GO:0015171	Amino acid transmembrane transporter activity	8.63E-06	8	*Ctns, Slc7a11, Slc16a10, Slc38a2, Slc7a2, Slc25a15, Slc7a1, Slc25a29*
GO:0005342	Organic acid transmembrane transporter activity	1.06E-05	11	*Slc25a32, Slc7a2, Slc13a4, Slc25a15, Slc17a2, Slc17a4, Ctns, Slco1b2, Slc26a1, Slc16a10*……
GO:0046943	Carboxylic acid transmembrane transporter activity	1.06E-05	11	*Slc25a32, Slc7a2, Slc13a4, Slc25a15, Slc17a2, Slc17a4, Ctns, Slco1b2, Slc26a1, Slc16a10*……
GO:0015179	L-amino acid transmembrane transporter activity	1.21E-05	7	*Ctns, Slc7a11, Slc38a2, Slc7a2, Slc25a15, Slc7a1, Slc25a29*
GO:0031625	Ubiquitin protein ligase binding	2.68E-05	15	*Gabarapl1, Fzd5, Erbb2, Per1, Ppargc1a, Cdkn1a, Map1lc3b, Cbs, Myc, Usp2*……
GO:0044389	Ubiquitin-like protein ligase binding	4.65E-05	15	*Gabarapl1, Fzd5, Erbb2, Per1, Ppargc1a, Cdkn1a, Map1lc3b, Cbs, Myc, Usp2*……
GO:0048037	Cofactor binding	6.40E-05	20	*Hao1, Gldc, Oat, Hacl1, Hbe1, Hbg1, Gpd1, Gstm2, Maob, Fmo3*……
GO:0020037	Heme binding	1.03E-04	10	*Hbe1, Nr1d1, Cyp2c13, Hbg1, Cyp3a9, Cbs, Hbz, Cyp2j10, Cyp2c22, Cyp21a1*
**(C) Top 4 GO terms for the CC**				
GO:0005777	Peroxisome	3.52E-05	10	*Ehhadh, Hao1, rat, Hacl1, Acot4, Fabp1, Acsl1, Pxmp4, Hsdl2, Amacr*
GO:0042579	Microbody	3.52E-05	10	*Ehhadh, Hao1, rat, Hacl1, Acot4, Fabp1, Acsl1, Pxmp4, Hsdl2, Amacr*
GO:0009331	Glycerol-3-phosphate dehydrogenase complex	2.20E-02	2	*Gpd2, Gpd1*
GO:0005811	Lipid droplet	2.71E-02	5	*Cidec, Pnpla2, Plin2, Hsd3b7, Nsdhl*

### KEGG Pathway Analysis of DEGs

**Figure 4:**
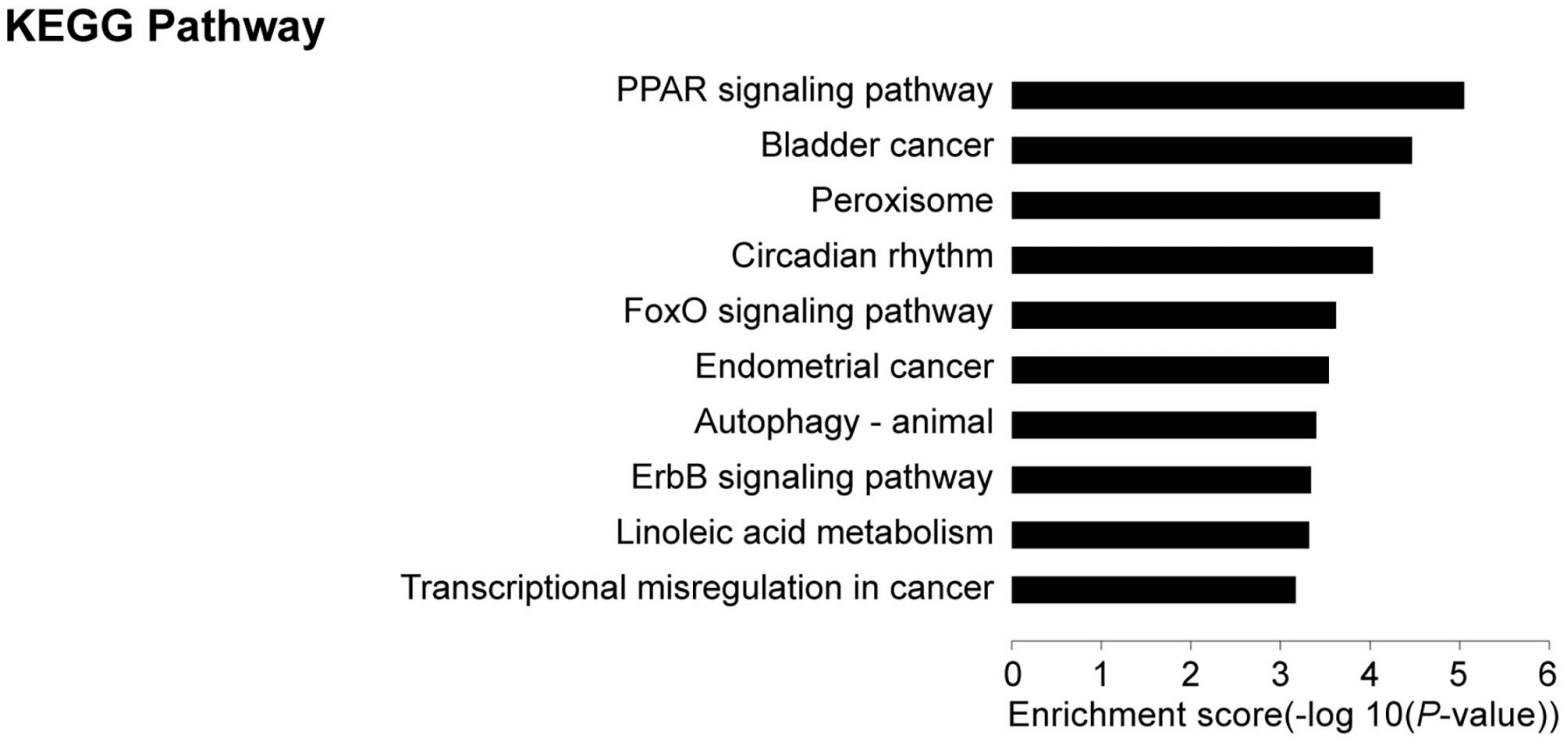
Results of KEGG analysis: Top 10 KEGG pathways in IUGR compared with AGA.

KEGG pathway analyses revealed that, in comparison with controls, target genes were enriched in 21 pathways, including PPAR (19 genes), FoxO (9 genes), autophagy—animal (9 genes), ErbB (7 genes), adipocytokine (6 genes), JAK-STAT (8 genes), and glucagon (8 genes) signaling pathways among others. The top 10 pathways are presented in Figure 4. These core pathways and their related genes are shown in Table 3.

**Table 3:** The core pathways analysis for DEGs between IUGR and AGA.

**Term**	**KEGG pathway**	***P*-value**	***N***	**Genes**
rno03320	PPAR signaling pathway	9.00E-06	19	*Aqp7, Gk, Rxrg, Fabp1, Fabp2, Fabp4, Fabp7, Slc27a5, Angptl4, Plin2*……
rno04068	FoxO signaling pathway	2.43E-04	9	*Cdkn1a, Egfr, Egf, Il10, Sgk1, Gadd45b, Gabarapl1, Prkaa2, Bnip3*
rno04140	Autophagy—animal	3.95E-04	9	*Ddit4, Bcl2l1, Dapk1, Uvrag, Rb1cc1, Prap1, Gabarapl1, Prkaa2, Bnip3*
rno04012	ErbB signaling pathway	4.58E-04	7	*Nrg1, Cdkn1a, Cblb, Egfr, Erbb2, Myc, Egf*
rno00591	Linoleic acid metabolism	4.84E-04	5	*Cyp3a9, Cyp2c22, Cyp2c13, Cyp2j10, Pla2g12a*
rno04920	Adipocytokine signaling pathway	1.06E-03	7	*Acsl4, Npy, Slc2a1, Nfkbia, Prkaa2, Ppargc1a, rxrg*
rno04066	HIF-1 signaling pathway	2.33E-03	7	*Cdkn1a, Ifngr1, Pfkfb3, Egfr, Erbb2, Slc2a1, Egf*
rno04630	JAK-STAT signaling pathway	3.08E-03	8	*Cdkn1a, Ifngr1, Egfr, Myc, Bcl2l1, Il6st, Egf, Il10*
rno04060	Cytokine–cytokine receptor interaction	3.45E-03	11	*Cxcl2, Ifngr1, Il1r2, Il6st, Il10, Il18, Tnfrsf12a, Il17rb, Relt, Il1rn*……
rno04922	Glucagon signaling pathway	4.50E-03	8	*Sik1, Ppargc1a, Prkaa2, Pde3b, Slc2a1, Fbp1, Ldha, Gcgr*

**Figure 5:**
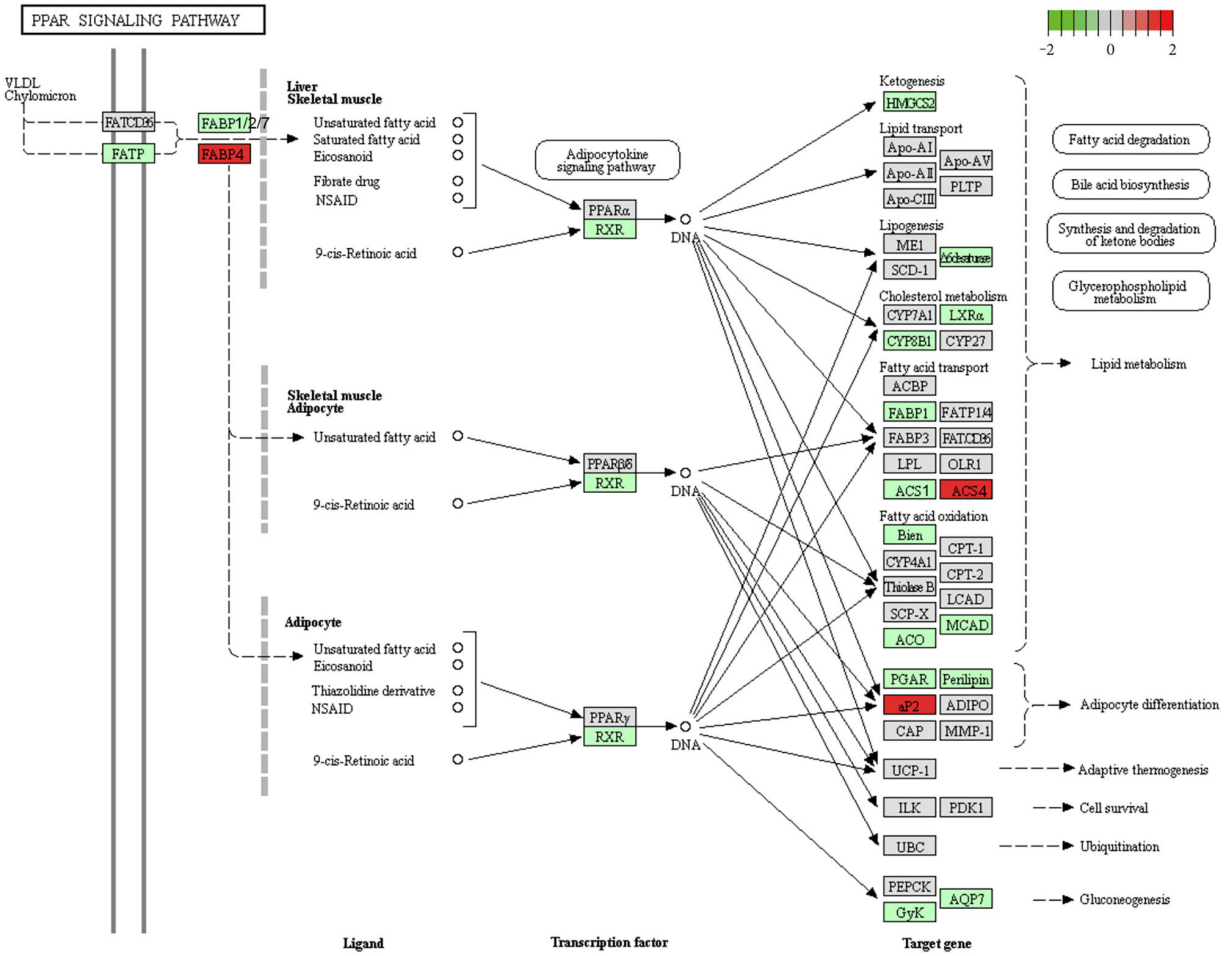
Downregulation of the PPAR signaling pathway in IUGR. We obtained the original Figure 5 from the KEGG website (https://www.kegg.jp/kegg-bin/show_pathway?map03320), and we made some modifications.

In the PPAR signaling pathway, FABP family (elevated *Fabp4* and suppressed *Fabp1, Fabp2*, and *Fabp7*), ACSL family (upregulated *Acsl4* and downregulated *Acsl1*) (Figure 5), and adipocytokine signaling (upregulated *Npy, Slc2a1, Nfkbia, Prkaa2*, and *Ppargc1a* and downregulated *Rxrg*) showed obvious differential expression. The findings indicate that the PPAR signaling pathway might be the most significant pathway in the occurrence of IUGR-caused metabolic syndrome.

These significantly enriched pathways indicated that six directly interacting genes with the glycometabolism signaling pathway were selected from multiple networks in KEGG pathways (Table 4). Finally, integrated bioinformatics pipeline related to study curation, GeneRIF as well as publications under “Related Articles” in PubMed showed that six genes associated with glycometabolism signaling pathways should be investigated further.

**Table 4:** 6 DEGs associated with glycometabolism signaling pathway in IUGR.

**Gene symbol**	**Gene description**	***P*-value**
*Ppargc1a*	Peroxisome proliferator-activated receptor gamma, coactivator 1 alpha	1.71E-03
*Prkaa2*	Protein kinase, AMP-activated, alpha 2 catalytic subunit	4.79E-05
*Slc2a1*	Solute carrier family 2 (facilitated glucose transporter), member 1	6.83E-04
*Acsl4*	Acyl-CoA synthetase long-chain family member 4	6.87E-03
*Rxrg*	Retinoid X receptor gamma	4.85E-05
*Gcgr*	Glucagon receptor	4.24E-03

### DEGs and Core Genes in the Interaction Network

**Figure 6:**
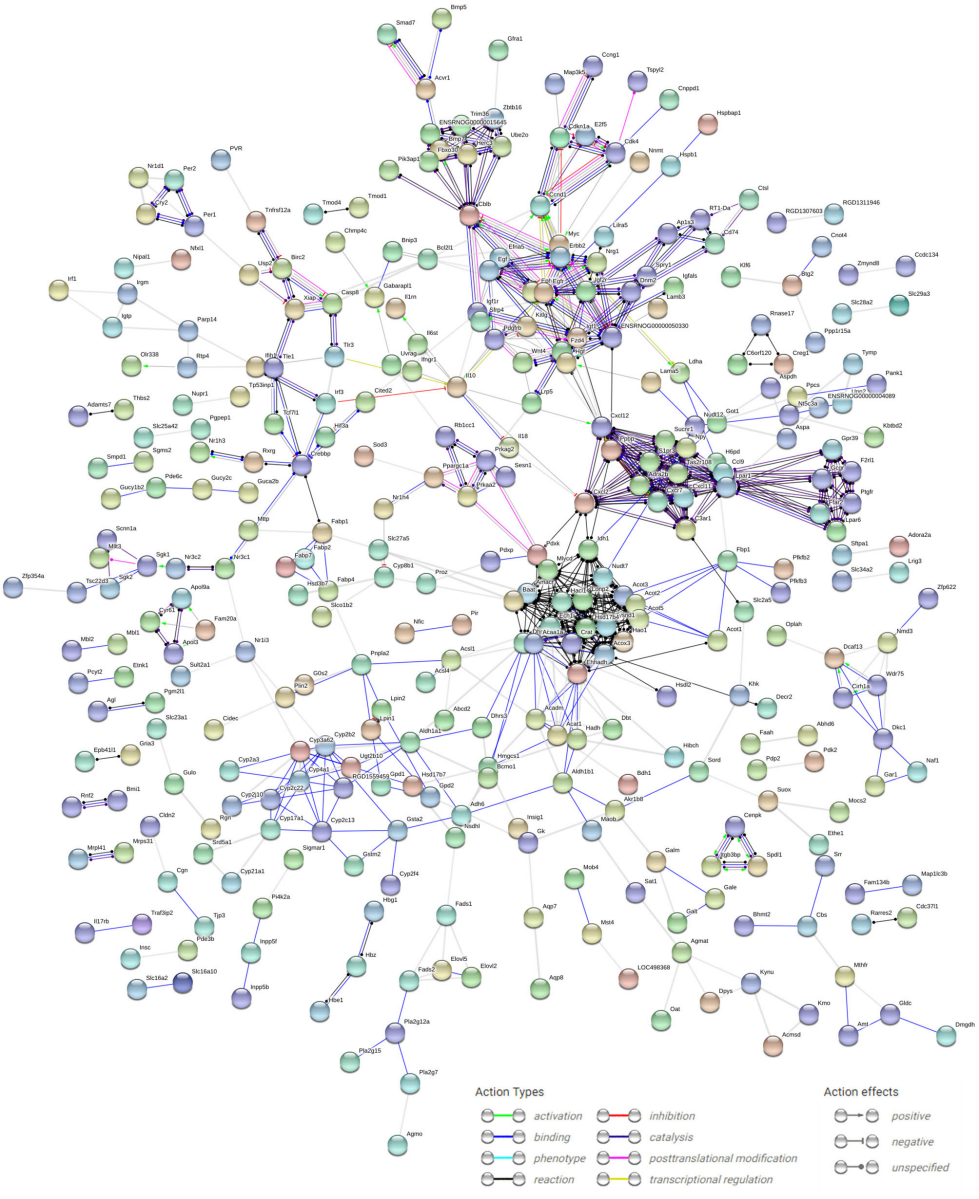
PPI network analysis of DEGs in IUGR. The dots indicate individual differentially expressed genes, and the lines between any nodes represent the interrelations of those proteins. The PPI network was established using cut-off values of confidence score >0.7 using default online parameter settings. We visualized the PPI network analysis of DEGs from the STRING database (version 10.5; http://string-db.org/).

According to data from the STRING database, the gene interaction network had 750 edges and 694 nodes (Figure 6). The nodes denoted DEGs, while edges denoted interactions among DEGs. These genes were analyzed using Network Analyzer in Cytoscape software, after which core genes were ranked based on projected scores. *Ehhadh, Gk, Slc2a1, Casp3, Fabp2, Fabp1, Npy, Prkaa2, Rxrg, Pfkb3, Sgk1, Gadd45b, Nr1h4, Galm, Gale, Hao1, Gcgr, Btg2, Nr1i3*, and *Egfr* were among the top 20 high-degree hub nodes. Among these, *Prkaa2, Slc2a1, Rxrg, Gcgr, Fabp1, Fabp2, Gk*, and *Sgk1* are associated with glycometabolism development as well as progression. This result suggested that the changes at glycometabolism levels contained in IUGR liver might affect metabolic syndrome.

### Gene Expression Validation by RT-QPCR

**Figure 7:**
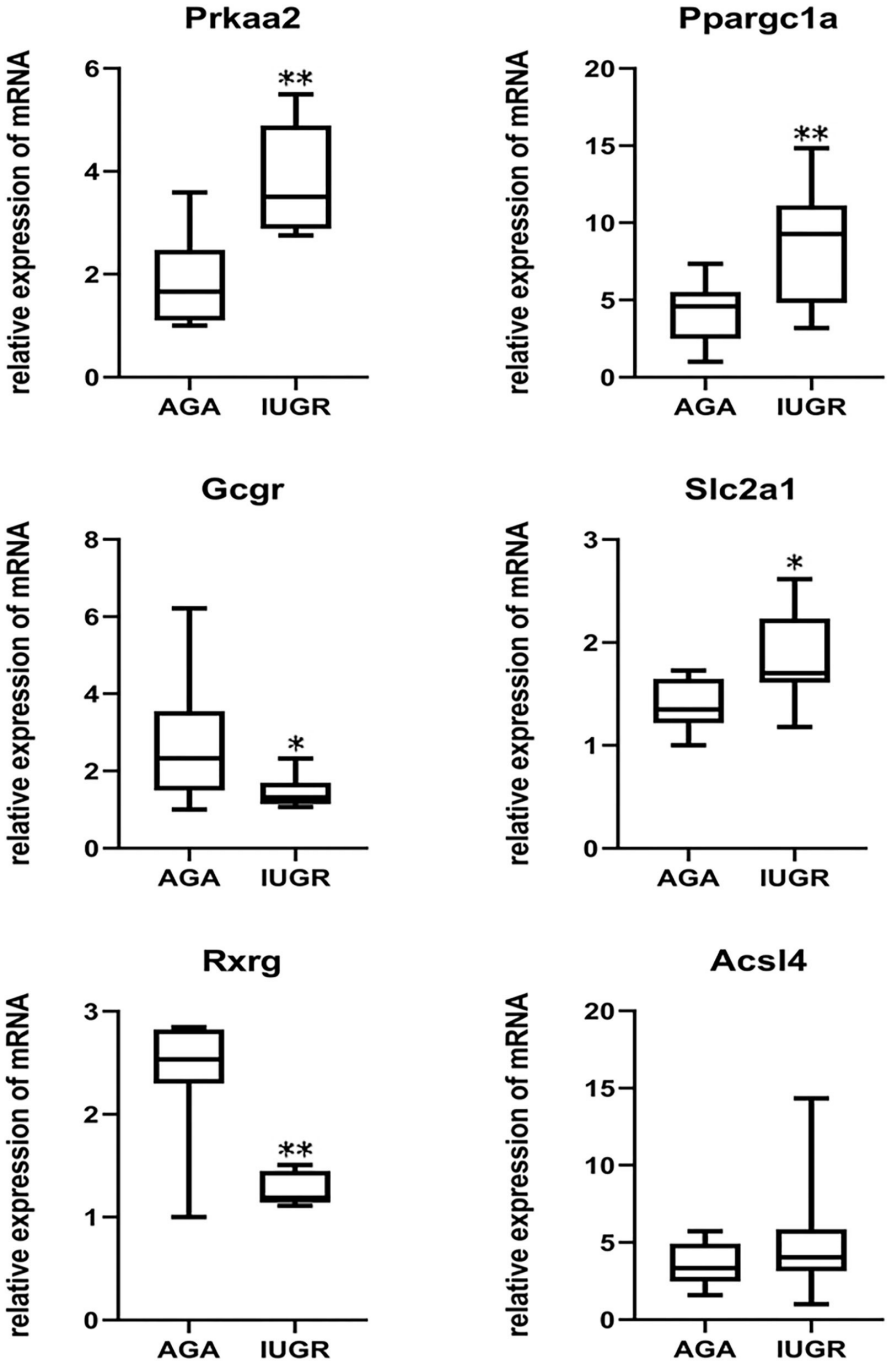
Validation of DEGs by RT-qPCR analysis. β*-actin* as an internal control. *Prkaa2* (IUGR: 3.81 ± 0.97; AGA: 1.86 ± 0.84)*, Ppargc1a* (IUGR: 8.72 ± 3.74; AGA: 4.21 ± 1.89)*, Gcgr* (IUGR: 1.47 ± 0.39; AGA: 2.74 ± 1.55)*, Slc2a1* (IUGR: 1.84 ± 0.42;AGA: 1.38 ± 0.24)*, Rxrg* (IUGR: 1.26 ± 0.15; AGA: 2.39 ± 0.56) and *Acsl4* (IUGR: 5.18 ± 3.75; AGA: 3.50 ± 1.33) mRNA levels were analyzed in samples of IUGR, compared with AGA. Data indicate relative expression following normalization (*P < 0.05, **P < 0.01).

Because IUGR had been considered as an important source of metabolic syndrome, we identified six glycometabolism-associated genes from microarray assays for validation (Table 4). They were *Ppargc1a, Prkaa2, Slc2a1, Acsl4, Rxrg*, and *Gcgr*, associated with different signaling pathways. As presented in Figure 7, expression levels of *Prkaa2, Ppargc1a*, and *Slc2a1* were markedly higher in IUGR than AGA (*P* = 0.030, 0.016, and 0.006). Levels of *Rxrg* and *Gcgr* were markedly lower in IUGR than AGA (*P* = 0.000003 and 0.018). *Acsl4* expression levels were elevated in IUGR than AGA, but without marked differences (*P* = 0.122). Our RT-qPCR findings were in agreement with microarray findings, confirming the reliability of the microarray data.

## Discussion

The inner molecular mechanism of adult metabolic syndrome caused by IUGR is poorly understood. The applications of gene expression microarrays in disease research are attributed to transcriptional changes that provide a sensitive and robust way for understanding disease mechanisms and their complications. Previously, microarray analyses were used to evaluate the underlying mechanisms of hypertension (26), glioblastomas (27), pulmonary hypertension (28), and so on in patients or animal models. Therefore, performing transcriptional microarray analyses through rat liver to identify variations in gene expression signatures of IUGR is important. In our study, we used gene microarray to identify different gene expressions between IUGR and AGA rat livers. We found 815 DEGs, among which 347 genes were elevated while 468 were suppressed. These results elucidate on the metabolic syndrome caused by IUGR.

Genes such as *Fam134b, Upp2, Aspa, Gadd45b*, and *Prkaa2* were significantly upregulated and *Paqr9, Fam82a1, Ifi47, Paqr7, G0s2*, and *Cyp4a1* were markedly suppressed in IUGR, relative to AGA. Remarkably, a wide range of significant DEGs in IUGR were related to lipid metabolism and glycometabolism. This finding supported our view that the change of lipid metabolism and glycometabolism in IUGR liver played critical roles in the metabolic syndrome. RT-qPCR analysis further verified our results. The results showed that among the six glycometabolism-related genes, three were upregulated, two were downregulated, and one tended to upregulate in IUGR compared with AGA, which were correlated with those obtained by microarray data. The DEGs between IUGR and AGA, particularly those of lipid metabolism- and glycometabolism-associated genes, further supported a special significance of IUGR in metabolic syndrome pathogenesis.

Similar bioinformatics analysis in other tissues of IUGR model revealed that many metabolism-related genes were expressed differently. Using microarray profiling, Zhang and Chen et al. (29) found that DEGs, such as *FoxO1, Pdx1*, and *MafA*, might be potential targets for the development of diabetes mellitus of IUGR. Through three gene expression profiling datasets and the verification of RT-qPCR, Madeleneau et al. (30) found that *Lep, Igfbp1*, and *Rbp4* might play important roles in energy production and metabolism. Freije et al. (31) reported that the DEGs were *Ccrn4lb, Per1, Per2, Per3, Nr1d1, Npas2, Arntl, Igfals, Nrep, Apol3, Got1*, and so on in IUGR liver compared to AGA at 21 days of age. Our study found *Igfbp1* was upregulated, and *Per1, Per2, Igfals, Nrep, Got1*, and *Apol3* expression were changed in IUGR.

In this study, GO analysis revealed that the DEGs were highly involved in the biological process related to small molecule metabolic process, fatty acid metabolic process, monocarboxylic acid metabolic processes, and lipid metabolic process. These findings indicated a pathophysiologic link between IUGR and fatty metabolism. Notably, many genes in the PPAR signaling pathway, such as upregulated *Fabp4* and *Acsl4* and downregulated *Fabp1, Gk, Angptl4, Ehhadh*, and *Acsl1*, were related to metabolism. *Angptl4* is a kind of adipokine involved in lipid metabolism, because of its wide expression in liver and adipose tissue (32). But recent studies indicated that *Angptl4* has a role in various metabolic as well as non-metabolic disorders and is particularly important in several energy homoeostasis aspects (33). At the same time, accumulating evidence associated *Angptl4* directly with the risk of atherosclerosis and T2D (34). Herrera et al. (35) found that maternal plasma *Angptl4* was decreased in gestational diabetes mellitus (GDM), which was consistent with our findings in our IUGR model. Recent studies also reported that the variation in the *Ehhadh* was related to not only non-alcoholic fatty liver disease (NAFLD) but also to T2D, central obesity, and WHO-defined metabolic syndrome (36). Thus, DEGs in small molecule metabolic process in IUGR might be the main molecular event that caused metabolic syndrome. Expecting DEGs in fatty acid metabolic as well as lipid metabolic processes, our microarray results revealed that DEGs were also involved in the biological function such as the coenzyme metabolic process (*Pfkfb3, Ppargc1a, Pdxk, Rgn*) and nucleobase-containing small molecule metabolic process (*Gucy2c, Elovl5, Slc25a33, Acot5*). As an example, in our study *Ppargc1a*, a major transcription factor in the regulation of metabolism as well as energy homeostasis, was markedly elevated in the IUGR liver. *Ppargc1a* played a vital role in glucose transport in skeletal muscle (37) and pancreatic beta cells (38) and coactivated the cholesterol 7-α-hydroxylase (*Cyp7a1*) gene that encoded an enzyme vitally needed for cholesterol metabolism (39, 40). Accordingly, molecular events related to the coenzyme metabolic process might be vital biological processes during the metabolic syndrome caused by IUGR.

In this study, GO CC analysis revealed that DEGs were highly enriched in peroxisome, microbody, glycerol-3-phosphate dehydrogenase complex, and lipid droplet. As an example, *Crat*, a mitochondrial matrix enzyme that promoted glucose disposal, was significantly decreased in the IUGR liver. *Crat* was reported to be significantly reduced in humans with T2D. In addition, *Crat*^*M*−/−^ mice fed a low-fat diet gained total body weight and fat pad mass at similar rates as control littermates (*Crat*^*fl*/*fl*^); however, when changed to a high-fat diet, *Crat*^*M*−/−^ mice had a greater body weight and fat mass than the control group. Interestingly, despite normal body weight when feeding on the low-fat diet, blood glucose levels were higher in the *Crat*^*M*−/−^ both at baseline and throughout an intraperitoneal glucose tolerance test. High-fat feeding further exacerbated the glucose intolerant phenotype of *Crat*^*M*−/−^ mice (41). Whether *Crat* played an indispensable role in defending whole body glucose homeostasis and how it mediated IUGR-induced metabolic syndrome needed further investigation.

IUGR might come from reduced placental nutrient transfer or insufficient maternal nutrient supply, causing suppressed placental as well as fetal growth; therefore, the need for investigating the nutrient transporters was necessary. Glucose transporters were vital for maintaining glucose supply (42). Recent studies have shown that the glucose transporter GLUT1 levels are increased in smooth muscle cell (SMC) in proximity to atherosclerotic lesions. Cytokines, including TNF-α, secreted by lesioned arteries, promote *Glut1* levels in SMCs, thereby increasing the levels of CCL2 by enhancing glycolysis and the polyol pathway. In addition, *Glut1* overexpressions in SMCs, but not in myeloid cells, accelerated the development of larger, more advanced lesions in a mouse model of metabolic syndrome (43). In this study, the analysis of GO MF revealed that DEGs mainly enriched in organic anion transmembrane transporter activity such as upregulated *Ctns, Slc2a1, Slc25a32, Slc7a11, Slc16a10, Slc38a2, Slc7a2, Slc25a15, Slc7a1*, and *Slc25a29* and downregulated *Slc13a4, Slc17a2, Slc17a4, Slc26a1, Slc16a7, Slc25a42*, and *Slc25a23*. The findings indicated that the change of transmembrane transporter-related regulatory genes might be an important event in the metabolic syndrome induced by IUGR.

KEGG analysis revealed that the DEGs participated in more than 25 pathways. Some of these well-known pathways, such as the PPAR, FoxO, adipocytokine, and glucagon signaling pathways, were found to play essential roles in metabolic syndrome. The results also showed that DEGs were mainly involved in the PPAR signaling pathway, including FABP family genes (elevated *Fabp4* and suppressed *Fabp1, Fabp2*, and *Fabp7*) and the ACSL family gene (elevated *Acsl4* and suppressed *Acsl1*). FABPs, a family of lipid chaperones, were correlated with metabolic syndromes, obesity, and atherosclerosis. Studies on FABPs revealed that the plasma levels of *Fabp4* were elevated in metabolic syndrome patients (44, 45) and the *Fabp1* null mice showed sex- and age-dependent weight gain as well as increased fat tissue mass (46). In line with previous studies, we found an altered FABP family gene in IUGR. Except for the FABP family gene, ACSLs also play a vital role in fatty acid metabolism. *Acsl4* was reported to be upregulated in patients with NAFLD (47). Here, *Acsl4* significantly increased in the IUGR compared with AGA, which indicated that upregulated *Acsl4* might be involved in the development of metabolic syndrome. Thus, the PPAR signaling pathway might be an important pathway during the IUGR-induced metabolic syndrome.

In conclusion, we identified 815 DEGs between IUGR and AGA, including 347 upregulated DEGs and 468 downregulated DEGs. It provided some associated key genes and pathways to understand the molecular mechanisms in IUGR-induced metabolic syndrome from GO and KEGG pathway analyses such as *Angptl4, Ehhadh, Ppargc1a, Crat, Slc2a1, Fabps, Acsls*, and so on, as well as the PPAR signaling pathway, which had an essential role in IUGR. Thus, our results might help to clarify the pathogenesis of metabolic syndrome caused by IUGR at the molecular level and provide new insight into early metabolic syndrome prevention as well as treatment. However, due to limitations of this study, additional experiments were needed to elucidate on the molecular mechanisms in IUGR-induced metabolic syndrome.

## Data Availability Statement

The original contributions presented in the study are included in the article/Supplementary Material, further inquiries can be directed to the corresponding author.

## Ethics Statement

The animal study was reviewed and approved by the Committee on the Ethics of Animal Experiments of the Zhejiang University.

## Author Contributions

LD responded for the design of the study and validation analysis. ZS participated in the design of the study, performed the bioinformatic analyses, and drafted the manuscript. WZ took part in the establishment of intrauterine growth retardation rat model, the sample selection, and the gene expression validation by RT-qPCR. All authors have read and approved the final manuscript.

## Funding

This work was supported by the Natural Science Foundation of Zhejiang Province (Grant Numbers LY17H040001, LQ18H070001) and the National Natural Science Foundation of China (Grant Numbers 81000267, 81471480).

## Conflict of Interest

The authors declare that the research was conducted in the absence of any commercial or financial relationships that could be construed as a potential conflict of interest.

## Publisher's Note

All claims expressed in this article are solely those of the authors and do not necessarily represent those of their affiliated organizations, or those of the publisher, the editors and the reviewers. Any product that may be evaluated in this article, or claim that may be made by its manufacturer, is not guaranteed or endorsed by the publisher.
