# Supplementary material for: Analysis of Gene Expression Profiles in the Liver of Rats With Intrauterine Growth Retardation
Source: Front Pediatr. 2022 Mar 7;10:801544. doi: 10.3389/fped.2022.801544 (PMC8934861; doi:10.3389/fped.2022.801544)
Supplement: Supplementary file 1 [file Data_Sheet_1.docx]

**Analysis of gene expression profiles in the liver of rats with intrauterine growth retardation**

**Zheng Shen^1,3^, Weifen Zhu^4^, Lizhng Du^2,3*^**

^1^Department of Clinical laboratory, Zhejiang University School of Medicine Children's Hospital, Hangzhou, China

^2^Department of Neonatology, Zhejiang University School of Medicine Children's Hospital, Hangzhou, China

^3^National Clinical Research Center for Child Health, Hangzhou, China

^4^Department of Endocrinology, Zhejiang University School of Medicine Sir Run Run Shaw Hospital, Hangzhou, China

***Correspondence:**

Lizhong Du

E-mail: dulizhong@zju.edu.cn

**S Table 1. Weight and sex information of pregnant rats and their offspring**

| Mother’s number | mother’s Pre-pregnancy weight | The number of neonate | male | female | Normal body weight | <2SD |
| --- | --- | --- | --- | --- | --- | --- |
| C1 | 273g | 13 | 7 | 6 | 13 | 0 |
| C2 | 270g | 13 | 5 | 8 | 13 | 0 |
| C3 | 268g | 16 | 9 | 7 | 16 | 0 |
| C4 | 293g | 14 | 8 | 6 | 14 | 0 |
| C5 | 252g | 14 | 6 | 8 | 14 | 0 |
| C6 | 267g | 15 | 10 | 5 | 15 | 0 |
| I1 | 261g | 13 | 6 | 7 | 0 | 13 |
| I2 | 251g | 14 | 6 | 8 | 0 | 14 |
| I3 | 266g | 12 | 7 | 5 | 1 male and 1 female | 10 |
| I4 | 280g | 13 | 6 | 7 | 0 | 13 |
| I5 | 279g | 13 | 5 | 8 | 1 male | 12 |
| I6 | 260g | 15 | 6 | 9 | 0 | 15 |

There was no significant difference in mother’s body weight of IUGR and control (P=0.56).

**S Table 2. Primer sequences for RT-qPCR**

| Gene | Accession# | Direction | Primer Sequences（5’→3’） | Size（bp） |
| --- | --- | --- | --- | --- |
| Ppargc1a | [NM_031347](https://www.ncbi.nlm.nih.gov/nuccore/NM_031347.1) | Forward  Reverse | ACCAAACCCACAGAGAACAG  GGGTCAGAGGAAGAGATAAAGTTG | 124 |
| Slc2a1 | [NM_138827](https://www.ncbi.nlm.nih.gov/nuccore/NM_138827.1) | Forward  Reverse | CCCTGCAGTTCGGCTATAAC  GAGTGTGGTGAGTGTGGTG | 125 |
| Prkaa2 | [NM_023991](https://www.ncbi.nlm.nih.gov/nuccore/NM_023991.1) | Forward  Reverse | CCAGAGCAAACCATACGACA  TCACGTAATTGCCAGTCACTG | 131 |
| Gcgr | [NM_172091](https://www.ncbi.nlm.nih.gov/nuccore/NM_172091.2) | Forward  Reverse | GAGAAGTGGAAGCTCTATAGTGAC  GCAGGAAATGTTGGCAGTG | 144 |
| Rxrg | [NM_031765](https://www.ncbi.nlm.nih.gov/nuccore/NM_031765.1) | Forward  Reverse | ACCAAGCAGAAGTATCCAGAAC  TCCATGAGGAAGGTGTCAATG | 149 |
| Acsl4 | [NM_053623](https://www.ncbi.nlm.nih.gov/nuccore/NM_053623.1) | Forward  Reverse | TTGGCTACTTACCTTTGGCTC  AATCACCCTTGCTTCCCTTC | 141 |
| Actb | [NM_031144](https://www.ncbi.nlm.nih.gov/nuccore/NM_031144.3) | Forward  Reverse | CACTTTCTACAATGAGCTGCG  CTGGATGGCTACGTACATGG | 148 |

**
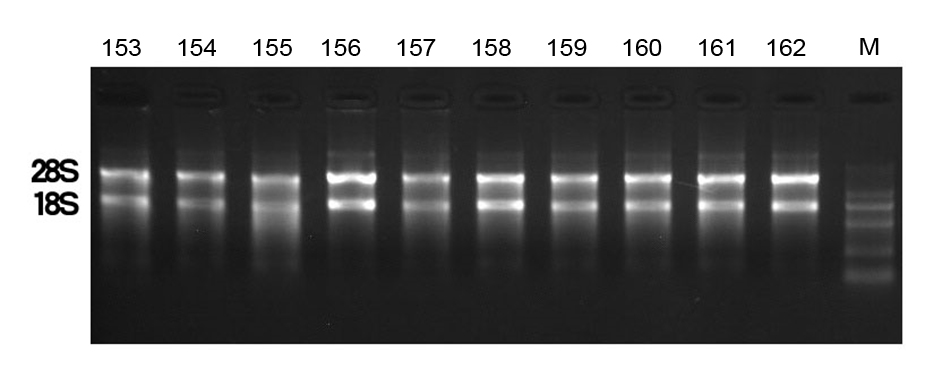
**

**S Fig 1. Total RNA electrophoretogram of rats liver tissue in IUGR and AGA**
